# First Reported Case of Fulminant Weil's Disease in South Khorasan, Eastern Iran: Diagnostic Challenges in a Non‐Endemic Region and Implications for Military Deployment

**DOI:** 10.1002/ccr3.72949

**Published:** 2026-06-22

**Authors:** Zohreh Azarkar, Anahita Arian, Motahareh Mahi‐Birjand, Parvin Askari

**Affiliations:** ^1^ Infectious Diseases Research Center Birjand University of Medical Sciences Birjand Iran; ^2^ Department of Internal Medicine, School of Medicine, Cardiovascular Diseases Research Center Birjand University of Medical Sciences Birjand Iran

**Keywords:** acute kidney injury, Iran, jaundice, leptospirosis, Weil's disease

## Abstract

Weil's disease should be considered even in non‐endemic regions when compatible clinical findings are accompanied by relevant exposure history. This case is epidemiologically notable as the first documented report from South Khorasan Province, highlighting the diagnostic challenges of leptospirosis in non‐endemic settings and the potential risk of disease transmission associated with military deployment from endemic to non‐endemic regions.

## 1 Introduction

Leptospirosis is a zoonosis caused by Leptospira species, particularly *Leptospira interrogans* , and is widely distributed worldwide. The reservoirs are primarily rodents, mainly rats. The infection in humans is acquired when people come in contact with urine from infected animals, contaminated ground or water, or infected food or drinking water, or through cuts, abrasions, and mucous membranes [1].

The disease can present with a wide range of symptoms, from mild or subclinical influenza‐like illness to Weil's disease, a severe and deadly form that causes hemorrhage, renal and hepatic failure, jaundice, and, in rare cases, severe pulmonary hemorrhage syndrome. Early antibiotics are essential for early diagnosis and for lowering morbidity and mortality [2, 3, 4].

In Iran, leptospirosis is endemic in the northern areas of Gilan, Mazandaran, and Golestan, which are primarily used for agriculture and rice cultivation. Between 2009 and 2018, 3433 cases were reported in 17 provinces, resulting in a combined incidence rate of 4.8 cases per 100,000 people [5]. Although leptospirosis is well recognized in northern endemic provinces of Iran, reports from eastern Iran are lacking. Here, we report what is, to the best of our knowledge, the first documented case of fulminant Weil's disease in South Khorasan Province. Beyond the clinical presentation, this case is noteworthy because it illustrates the epidemiological importance of internal population movement, particularly military deployment from endemic to non‐endemic regions, and emphasizes the importance of exposure history in establishing diagnosis where clinician suspicion is naturally low.

## 2 Case History/Examination

A 20‐year‐old Iranian male soldier from Gonbad‐e Kavus, recently deployed for military service in South Khorasan (Birjand), presented to the emergency department in July with a seven‐day history of high‐grade continuous fever accompanied by chills, severe myalgia, headache, photophobia, nausea, vomiting, and diffuse abdominal pain. The patient had relocated from Golestan Province, a recognized endemic area for leptospirosis in northern Iran, to South Khorasan, where leptospirosis is not typically encountered in routine clinical practice.

He also reported hemoptysis, dark‐red–colored urine, and black stools (melena) during the course of illness. The patient complained of fatigue, dizziness, and persistent abdominal pain, which intensified in the later stages of his disease.

He was non‐diabetic, non‐hypertensive, a non‐smoker, and non‐alcoholic, with no history of contact with jaundiced individuals, blood transfusion, or drug abuse. From an epidemiological standpoint, about three to 4 weeks before his deployment to Birjand, he had worked barefoot and barehanded in rice paddy fields in Gonbad‐e Kavus, where he was directly exposed to surface water. His clinical symptoms began shortly after returning to Birjand.

On admission, his vital signs were as follows: blood pressure, 120/80 mmHg; pulse rate, 85/min (regular); respiratory rate, 18/min; and SpO_2_, 94% on room air. Physical examination revealed conjunctival suffusion, icterus, splenomegaly, epigastric tenderness, and hematuria.

**FIGURE 1:**
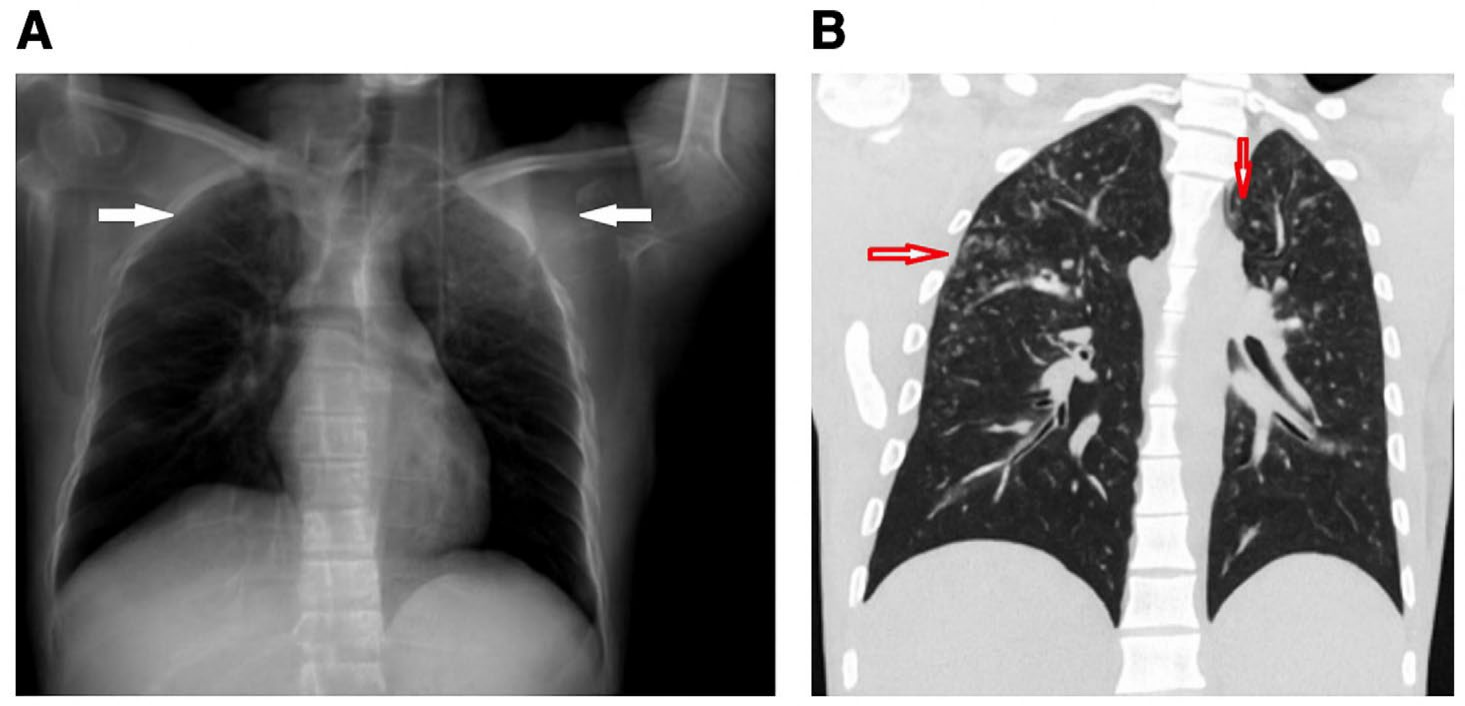
(A) Chest X‐ray showing bilateral apical opacities (white arrows). (B) Coronal High‐Resolution Computed Tomography (HRCT) of the thorax demonstrating micronodular ground‐glass opacities in the bilateral upper and lower lobes, more prominent in the posterior regions (red arrows).

Chest Computed Tomography (CT) demonstrated micronodular Ground‐Glass Opacities (GGO), more prominent in the posterior regions of both upper and lower lobes, with no pleural or pericardial effusion or significant lymphadenopathy (Figure 1). Abdominal ultrasonography revealed splenomegaly with normal liver echotexture and bile ducts, and no abnormal findings in the pancreas or kidneys. No free intraperitoneal fluid was detected.

## 3 Methods (Differential Diagnosis, Investigations, and Treatment)

A comprehensive diagnostic workup was initiated targeting both common and uncommon infectious agents, including Rickettsia and Leptospira species. Differential diagnoses considered included hemolytic uremic syndrome, malaria, dengue fever, brucellosis, and viral hepatitis.

Serial blood parameters are displayed in Table 1. Malaria parasite using peripheral blood smear microscopy, G6PD test, troponin test (TPI), HBsAg, anti‐HCV, IgM anti‐HAV, IgM anti‐HEV, and HIV was negative. The patient was diagnosed with metabolic acidosis based on Arterial Blood Gas (ABG) analysis, characterized by decreased pH, reduced bicarbonate (HCO3^−^), and a negative base excess (BE). The ABG analysis also revealed low oxygen saturation, ranging from 78% to 90%. Blood culture, Wright test, Coombs Wright test, and 2‐Mercaptoethanol test (2ME) all returned.negative results. Given a strong clinical suspicion, serum for IgM Leptospira and blood and urine samples were sent to the reference laboratory for molecular detection. Both C‐reactive protein (CRP) and ESR were high. Liver enzymes, Lactate Dehydrogenase (LDH), ALP, and CPK remained elevated up to the fifth day of hospitalization.

**TABLE 1:** Routine biochemical tests on day 1, day 3, day 5 & day 7.

Parameter	Day 1	Day 3	Day 5	Day 7	Reference range
TLC (/cmm)	10,900	12,900	10,300	7900	4000–11,000
Hb% (g/dL)	7.5	7.7	7.8	8.1	12–17.5
**DC**	N78L14M5E3	N67L26M3E4	N68L24M4E4	N64L28M5E3	Neutrophils: 40%–75% Lymphocytes: 20%–45% Monocytes: 2%–10% Eosinophils: 1%–6%
Platelet Count (/cmm)	0.27	0.50	1.27	1.59	150,000–450,000
Total bilirubin (mg/dL)	19.3	17.9	16.7	4.6	0.3–1.2
Direct bilirubin (mg/dL)	13.5	14.5	12.1	1.5	0.0–0.6
SGOT (U/L)	215	87.5	100	57	10–37
SGPT (U/L)	109	67.0	133	105	7–41
ALP (U/L)	317	321	250	—	< 270
Amylase (U/L)	165	113	—	—	< 95
Lipase (U/L)	74	17.0	—	—	13–60
CPK (U/L)	5011	435	110	100	< 174
LDH (U/L)	1141	679	718	482	240–480
Na^+^ (meq/L)	132	134	139	135	135–145
K^+^ (meq/L)	4.1	3.3	4.4	4.1	3.5–5.1
Urea (mg/dL)	119	159	103	29.0	15–45
Creatinine (mg/dL)	5.2	5.4	1.5	1.0	0.6–1.3
Urine WBC (/HPF)	10–15	6–8	0–1	0–1	0–5
Urine RBC (/HPF)	6–8	4–5	1–2	1–2	0–3
Urine protein	Pos	Neg	Neg	Neg	—
P‐time (sec)	11.8	11.9	12.0	12.4	10–13.0
INR	1.0	1.0	1.0	1.0	0.8–1.2
APTT (sec)	28	27.3	27.8	28.1	23–38
CRP (mg/L)	150	110	65	15	0–6
ESR (mm/h)	52	45	31	22	< 10

Abbreviations: ALP, Alkaline Phosphatase; APTT, Activated Partial Thromboplastin Time. CPK, Creatine Phosphokinase; CRP, C Reactive Protein. DC, Differential Count; ESR, Erythrocyte Sedimentation Rate; Hb, Hemoglobin; INR, International Normalized Ratio, LDH, Lactate Dehydrogenase; RBC, Red Blood Cell; SGOT, Serum Glutamic Oxaloacetic Transaminase; SGPT, Serum Glutamic Pyruvic Transaminase; TLC, Total Leukocyte Count; WBC, White Blood Cell.

Empirical therapy with ceftriaxone 1 g Intravenous (IV) every 12 h was commenced and maintained for 7 days, supplemented by supportive management with intravenous fluids.

Results of IgM Leptospira came on Day 3, and it was 29.2 U/mL (Negative < 9.0, Intermediate 9.0–11.0, Positive > 11.0). Real‐time PCR analysis was performed at the National Reference Laboratory for Zoonotic Diseases, affiliated with the Ministry of Health and Medical Education (MOHME), Iran. The results demonstrated positive detection of Leptospira spp. and pathogenic Leptospira DNA in urine samples. In whole blood, Leptospira spp. was detected, while pathogenic Leptospira was negative.

## 4 Conclusions and Results (Outcome and Follow‐Up)

These findings confirmed leptospiral infection, with urine showing a stronger positivity profile compared to blood. Abdominal pain, fever, and hemoptysis decreased on day seven. Jaundice was progressively diminished. On the tenth day, the patient was discharged. The patient's acute kidney injury improved with intravenous fluid therapy and supportive management, without the need for dialysis. A detailed clinical timeline according to CARE guidelines is presented in Table 2.

**TABLE 2:** Clinical timeline from symptom onset to discharge (based on CARE guidelines).

Day (relative to symptom onset)	Clinical event	Diagnostic tests	Interventions	Response
Day 1–7	Fever, myalgia, headache, nausea, abdominal pain	None (patient did not seek care)	None	Progressive worsening
Day 7 (day of admission)	Jaundice, hemoptysis, dark urine, melena, dizziness, fatigue	CBC: Hb 7.5, Plt 27,000; Cr 5.2, T.Bil 19.3; ABG: metabolic acidosis; Blood smear (malaria negative)	IV fluids, ceftriaxone started	Initial stabilization
Day 3 of hospitalization (Day 10 of illness)	Persistent fever, abdominal pain, oliguria	CBC: Plt 50,000; Cr 5.4, T.Bil 17.9; IgM Leptospira: 29.2 U/mL (positive); PCR: urine positive, blood partial	Continued ceftriaxone, IV fluids	Diuresis begins
Day 5 of hospitalization (Day 12 of illness)	Decreasing fever, improving abdominal pain	CBC: Plt 127,000; Cr 1.5, T.Bil 16.7	Continued ceftriaxone	Clinical improvement
Day 7 of hospitalization (Day 14 of illness)	Afebrile, hemoptysis resolved, jaundice decreasing	CBC: Plt 159,000, Cr 1.0, T.Bil 4.6; CRP 15	Ceftriaxone completed (7‐day course)	Marked improvement
Day 10 of hospitalization (Day 17 of illness)	No symptoms, fully ambulatory	Discharge labs: Cr 1.0, T.Bil normal		

## 5 Discussion

Leptospirosis is an emerging worldwide public health problem [6], yet this case presents unique challenges due to its occurrence in a non‐endemic region (South Khorasan, Eastern Iran) and its fulminant presentation with concurrent pulmonary, hepatic, renal, and hematologic involvement. The 7‐day delay from symptom onset to presentation is noteworthy and likely multifactorial: the patient's initial non‐specific flu‐like symptoms (fever, myalgia, headache) may have been mistaken for a self‐limiting viral illness; as an active duty soldier, he might have postponed seeking care; and the rarity of leptospirosis in eastern Iran (no prior reported Weil's disease cases in this region) lowered initial clinical suspicion.

The broad differential diagnosis included malaria, dengue, brucellosis, viral hepatitis, hemolytic uremic syndrome, and rickettsial diseases. Malaria was excluded via negative peripheral blood smear (performed on Day 1). Brucellosis was ruled out by negative Wright, Coombs Wright, and 2‐mercaptoethanol tests. Viral hepatitis (HAV, HBV, HCV, HEV) and HIV serologies were negative. The normal G6PD level excluded drug‐induced hemolysis. The persistent elevation of liver enzymes (SGOT 215 U/L, SGPT 109 U/L) with disproportionate hyperbilirubinemia (total bilirubin 19.3 mg/dL, direct 13.5 mg/dL) a ratio of direct to total ~0.7 indicated mixed hepatocellular and cholestatic injury, characteristic of leptospirosis rather than pure viral hepatitis. Conjunctival suffusion, present in this patient, is a highly specific clinical clue for leptospirosis (reported in 30%–40% of cases) and should have raised suspicion earlier.

The chest CT findings merit careful discussion. In leptospirosis, pulmonary hemorrhage syndrome occurs in 20%–70% of severe cases and carries a mortality rate of up to 50% when mechanical ventilation is required. The triad of hemoptysis, diffuse ground‐glass opacities (GGOs) on CT (representing alveolar hemorrhage), and falling hemoglobin (7.5 g/dL on admission, despite no overt bleeding source) strongly suggests pulmonary hemorrhage. The absence of pleural effusion or lymphadenopathy helps distinguish leptospirosis from tuberculosis or malignancy. The patient's hemoptysis resolved by Day 7 without requiring intubation—a favorable outcome given the severity of radiological findings.

Empirical ceftriaxone (1 g IV every 12 h for 7 days) was chosen over penicillin or doxycycline based on: (1) ceftriaxone's excellent activity against Leptospira spp. with once‐daily or twice‐daily dosing, (2) the need for broad empirical coverage given diagnostic uncertainty (covering possible community‐acquired pneumonia and other zoonoses), and (3) local antibiotic availability. While penicillin G (1.5 million units IV every 6 h) is considered first‐line in many guidelines, ceftriaxone is equally effective with a more convenient dosing schedule. Doxycycline was avoided initially due to concerns about gastrointestinal intolerance and the severity of illness requiring parenteral therapy. The 7‐day course is consistent with WHO recommendations for severe leptospirosis, though some experts extend treatment to 10–14 days in cases with multiorgan failure.

Despite presenting with severe thrombocytopenia (27,000/cmm on Day 1 a critical value increasing bleeding risk), acute kidney injury (creatinine 5.4 mg/dL on Day 3), metabolic acidosis, hyperbilirubinemia (19.3 mg/dL), and elevated CPK (5011 U/L indicating severe myositis), the patient recovered without dialysis or mechanical ventilation. Several factors explain this favorable trajectory: young age (20 years) with no comorbidities, absence of septic shock (blood pressure 120/80 mmHg on admission), early initiation of effective antibiotics (Day 1 of admission, albeit Day 7 of illness), and aggressive supportive care including intravenous fluids. The rapid decline in creatinine from 5.4 mg/dL (Day 3) to 1.5 mg/dL (Day 5) suggests pre‐renal acute kidney injury superimposed on acute interstitial nephritis, with preserved renal reserve allowing swift recovery. The platelet count normalized by Day 7 (159,000/cmm), consistent with the self‐limited nature of leptospira‐induced immune thrombocytopenia.

The PCR findings are enlightening, urine was positive for both Leptospira spp. and pathogenic Leptospira DNA, while whole blood was positive only for Leptospira spp. (negative for pathogenic Leptospira). This pattern indicates that bacteremia was clearing by the time of sampling (Day 3 of hospitalization/Day 10 of illness), while renal shedding persisted, a common finding as leptospires localize in renal tubules. Urine PCR may remain positive for weeks, whereas blood PCR becomes negative after the first week of illness. The IgM ELISA level of 29.2 U/mL (positive > 11.0) confirmed an acute immune response.

The patient was discharged on Day 10 without documented long‐term follow‐up. Given that leptospirosis can cause chronic kidney disease and sensorineural hearing loss, we recommend: (1) serum creatinine and urinalysis at 3 and 6 months post‐discharge to exclude chronic tubulointerstitial nephritis, (2) audiometry testing at 3 months, as hearing loss may be subclinical and permanent, and (3) blood pressure monitoring, as leptospirosis has been associated with new‐onset hypertension in some cohorts. The authors are attempting to contact the patient for 6‐month follow‐up data.

The novelty of this report does not primarily lie in the clinical manifestations, which were largely compatible with classical Weil's disease, including jaundice, acute kidney injury, thrombocytopenia, and pulmonary involvement. Rather, the significance of this case is epidemiological and operational.

First, to the best of our knowledge, this represents the first documented case of Weil's disease from South Khorasan Province, an eastern region of Iran not previously recognized as endemic for leptospirosis. Reporting cases from non‐endemic areas is important for disease surveillance and may indicate under‐recognition rather than true absence of disease.

Second, this case highlights a potential but underappreciated pathway for disease recognition in military settings. Personnel may acquire infection in endemic provinces and subsequently develop symptoms after deployment or relocation to non‐endemic areas, where clinicians may not initially consider leptospirosis in the differential diagnosis.

Third, the case underscores the diagnostic value of careful exposure history. In this patient, the history of barefoot exposure to rice paddies in Golestan before deployment was the critical epidemiological clue that prompted targeted testing and early empiric treatment. Without this information, diagnosis could have been delayed because the receiving center was located in a low‐suspicion geographic setting.

## 6 Conclusion

Diagnosis for any patient presenting with acute febrile illness, jaundice, renal impairment, and thrombocytopenia even without known occupational exposure or residence in endemic areas. The patient's history of rice field work in Gonbad‐e Kavus (an endemic region) 3 weeks prior to deployment to South Khorasan (non‐endemic) underscores the importance of eliciting travel history and remote exposures. Second, pulmonary hemorrhage syndrome in leptospirosis (manifested by hemoptysis, GGOs on CT, and falling hemoglobin) is a life‐threatening but potentially reversible complication if antibiotics are initiated promptly. Third, military health screening and deployment protocols should consider pre‐deployment counseling for soldiers moving between endemic and non‐endemic regions, emphasizing personal protective measures (boots, gloves) when exposed to potentially contaminated water sources. Finally, the favorable outcome despite severe laboratory abnormalities (platelets 27,000, creatinine 5.4, bilirubin 19.3) demonstrates that even delayed presentation (Day 7 of illness) does not preclude recovery with appropriate antibiotic and supportive care. Long‐term follow‐up for renal function and hearing is recommended for all severe leptospirosis survivors.

## Author Contributions

**Zohreh Azarkar:** conceptualization, project administration. **Anahita Arian:** investigation. **Motahareh Mahi‐Birjand:** visualization. **Parvin Askari:** writing – original draft, writing – review and editing.

## Funding

The authors have nothing to report.

## Disclosure

Patient Consent Declaration: The authors affirm that they have acquired the necessary patient consent papers. The patient has consented to the publication of his photos and other clinical information in the journal. The patient acknowledges that his name and initials will be kept confidential, and appropriate measures will be taken to protect his identity.

## Ethics Statement

The data collection process adhered to ethical guidelines and received approval from the university ethics committee, as indicated by the approval code “IR.BUMS.REC.1404.292”.

## Conflicts of Interest

The authors declare no conflicts of interest.

## Data Availability

The authors have nothing to report.
